# Exploring the potential of Brazilian Amazonian scorpion venoms: A comprehensive review of research from 2001 to 2021

**DOI:** 10.1016/j.toxcx.2023.100182

**Published:** 2023-12-29

**Authors:** Joel Ramanan da Cruz, Philippe Bulet, Cléria Mendonça de Moraes

**Affiliations:** a Post-graduation Program in Health and Biodiversity, Federal University of Roraima, Boa Vista, Roraima, Brazil; b Institute pour l’Avancée des Biosciences, CR Université Grenoble Alpes, Inserm U1209, CNRS UMR 5309, 38000, Grenoble, France; c Platform BioPark Archamps, ArchParc, 218 Avenue Marie Curie, 74160, Archamps, France; d Health Sciences Center and Post-graduation Program in Health and Biodiversity, Federal University of Roraima, Boa Vista, Roraima, Brazil

**Keywords:** Amazonian scorpions, Bioactive molecules, Biome, Toxins, Venom

## Abstract

The Amazon biome is home to many scorpion species, with around two hundred identified in the region. Of these, forty-eight species have been reported in Brazil so far and six of them are of medical importance: *Tityus apiacas, T. metuendus, T. obscurus, T. raquelae, T. silvestris*, and T*. strandi*. Three non-medically important species have also been studied: *Opisthanthuscayaporum*, *Brotheas amazonicus* and *Rhopalurus laticauda*. The venom of the scorpion *T. obscurus* is the most studied, followed by *O. cayaporum*. We aim to update the study of these Amazonian scorpion species. We will explore the harmful and beneficial properties of scorpion venom toxins and how they could be applied in drug development. This systematic review will focus on collecting and analyzing venoms from scorpions in Brazil. Only papers on Amazonian scorpion venom studies published between 2001 and 2021 (scientific articles, theses, and dissertations) were selected, based on the lists of scorpions available in the literature. Species found in the Amazon but not confirmed to be Brazilian were omitted from the review. Theses and dissertations were chosen over their derived articles. We found 42 eligible studies (13 theses, 27 articles and 2 patents) out of 17,950 studies and a basic statistical analysis was performed. The literature showed that *T. obscurus* was the most studied venom with 28 publications, followed by *O. cayaporum* with seven articles, *B. amazonicus* with four articles, *T. metuendus* with two article and *R. laticauda* with one article. No publication on the characterization of *T. silvestris* and *T. apiacas* venoms were found during the reviewed period, only the clinical aspects were covered. There is still much to be explored despite the increasing number of studies conducted in recent years. Amazonian scorpions have promising potential for pharmaceutical and clinical applications.

## Highlights

• Only a 5 of the 48 reported scorpions in the Brazilian Amazon have been studied for their venom.
• *Tityus obscurus* and *T. metuendus* are of medical importance.
• *Opisthacanthus cayaporum*, *Brotheas amazonicus*, *Rhopalurus laticauda* remain poorly studied.
• *T. obscurus* venom is the most researched, presumably because of its medical importance.

## Graphical abstract

**Figure:**
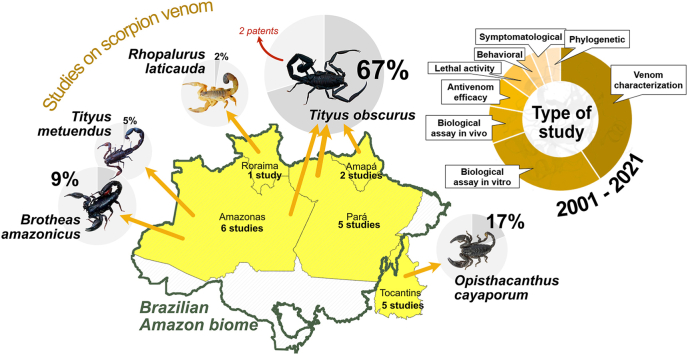

## 1 Introduction

With an estimated 1.8 million species of organisms, the Amazon is known for its unique and extensive biodiversity, high endemism, and its value as a source of genetic, chemical and ecological data, as well as raw materials for the industry and pharmaceutical laboratories (Sá et al., 2019). The biome covers nine Brazilian states (Acre, Amapá, Amazonas, Mato Grosso, Pará, Rondônia, Roraima, Tocantins, and part of Maranhão) representing 61 percent of the country's land area (approximately 5,217,423 km^2^). It contains a wide variety of ecosystems, human populations, cultures, and traditional communities (Martha-Júnior et al., 2011). The biome is also home to multiple scorpion species, some of which are medically relevant (Monteiro et al., 2019). Accidental envenomation from their stings makes scorpions a significant public health concern, with reports of hospitalizations and deaths worldwide (Abroug et al., 2020). Approximately 200 scorpion species have been recorded in the Amazon region. Martins et al. (2021) found forty-eight scorpions in the Brazilian Amazon, six of which are medically important: *Tityus apiacas*, *T. metuendus*, *T. obscurus*, *T. raquelae*, *T. silvestris*, and *T. strandi* (Borges et al., 2021). Scorpion venom is a complex mixture of compounds used for defense and prey capture (Quintero-Hernández et al., 2013). The venom contains a variety of compounds, such as neurotoxins that act on different ion channels through specific receptors. Venoms can be classified based on the three-dimensional structure of the toxins and the type of response elicited. In general, venom compounds are classified according to their nature/structure. Table 1 shows the most common venom compositions.

**Table 1:** Classification of venom compounds by nature and structure, according to Ahmadi et al. (2020) and de Oliveira et al. (2018).

Non-proteinaceous components	water, mucosa, nucleotides, mucopolysaccharides, lipids, metals, and inorganic compounds.
Proteinaceous components	*non-disulfide bridged peptides, NDBP*: small peptides with 13–56 amino acids with no predictable structure-function relationship.
	*cystine-stabilized alpha/beta scaffold peptides*: a disulfide bridge connects an α-helix to a double or triple-stranded β-sheet.
	*Short toxins*: with 23–64 residues, commonly potassium channel toxins
	*Long toxins:* with 55–76 residues, commonly sodium channel toxins;
	*Calcines*: a family of calcium channel-modulating peptides, which interestingly can pass through cell membranes without causing them to rupture.
	*Enzymes*: commonly hyaluronidases, phospholipases, glycosidases, nucleotidases, metalloproteases, serine proteases, ACE-like (angiotensin converting enzyme-like).
	*Other proteins:* CRISPs (cysteine-rich secretory proteins).

Scorpion toxins are grouped into different families according to their pharmacological targets: sodium, potassium, chloride and calcium channels (Cid-Uribe et al., 2020) and other cell membrane receptors (Hakim et al., 2015). Toxins that act on sodium channels are called NaTx. They are classified as α-NaTx if they bind to receptor site 3, or β-NaTx, if they bind to receptor site 4. Toxins that act on potassium channels are called KTx and are grouped into seven families: α-KTx, β-KTx, γ-KTx (Ergtoxins), δ-KTx, ε-KTx, κ-KTx (Hefutoxins), and λ-KTx. Calcines and Liotoxins are calcium-channel binding toxins (CaTx). Romero-Gutierrez and colleagues considered Omegascorpins as a new CaTx subfamily (Romero-Gutierrez et al., 2017). Toxins that act on chlorine channels are named ClTx with the single classification α-ClTx. Toxins that act on Transient Receptor Potential (TRP) channels are named TRPTx with the single classification α-TRPTx (Cid-Uribe et al., 2020). The venom of these arachnids is a mixture of proteins, peptides, nucleotides, and amines. It targets excitable and immunological cells, especially potassium, calcium, chlorine and sodium channels. An increasing number of studies have focused on their composition and bioactivity, suggesting their potential use in medical treatments and drug development (Ghosh et al., 2019). Scorpion venom toxins have been found to have several important pharmacological and insecticidal properties. These include analgesic, immunostimulatory, anticoagulant, antithrombotic, antimalarial, antiproliferative, anti-inflammatory, antiviral, anti-infectious, antiepileptic, antihypertensive, anti-osteoporotic, and antitumor effects (Ahmadi et al., 2020; Ghosh et al., 2019). An overview of scorpion venom studies is helpful to evaluate what we know, identify research gaps, and guide future investigations (Paul and Criado, 2020). The purpose of this literature review is to present and analyze studies on Amazonian scorpion venoms published between 2001 and 2021 in scientific articles or theses and dissertations available online, in order to understand the scientific advancements, challenges, and trends within this domain over the past two decades.

## 2 Overview of scorpion knowledge in Amazonia

We conducted a systematic review of publications from 2001 to 2021 that described studies on Amazonian scorpion venom. This was based on scorpion lists provided by Brazil and Porto (2010) and Borges et al. (2021). We excluded studies on species found in the Amazon, but not listed as Brazilian. Due to the greater amount of information, theses and dissertations were chosen over their derived articles. We included theses and dissertations in the literature review to provide an in-depth analysis of the results and to help contextualize the research. Future perspectives of this work are also detailed. To retrieve scientific papers published or available online, we consulted Google Scholar, the Brazilian Digital Library of Theses and Dissertations (BDTD, http://bdtd.ibict.br/vufind/), the *Catálogo de Teses e Dissertações da CAPES*, PubMed, the Virtual Health Library, the *Rede Iberoamericana de Innovación y Conocimiento Científico* - REDIB, the Networked Digital Library of Theses and Dissertations – NDLTD, the EBSCO Open Dissertations, Cochrane Library, the National Institute of the Industrial Property (INPI) and Espacenet using the following keywords in English and Portuguese: “peçonha”, “venom”, “scorpion venom”, “peçonha de escorpião”; “scorpion venom + amazon”; “Amazon scorpion”, “peçonha de escorpião + amazônia”; “scorpion venom characterization”, names of scorpion species and others related to the research.

### 2.1 Literature selection

**Fig. 1:**
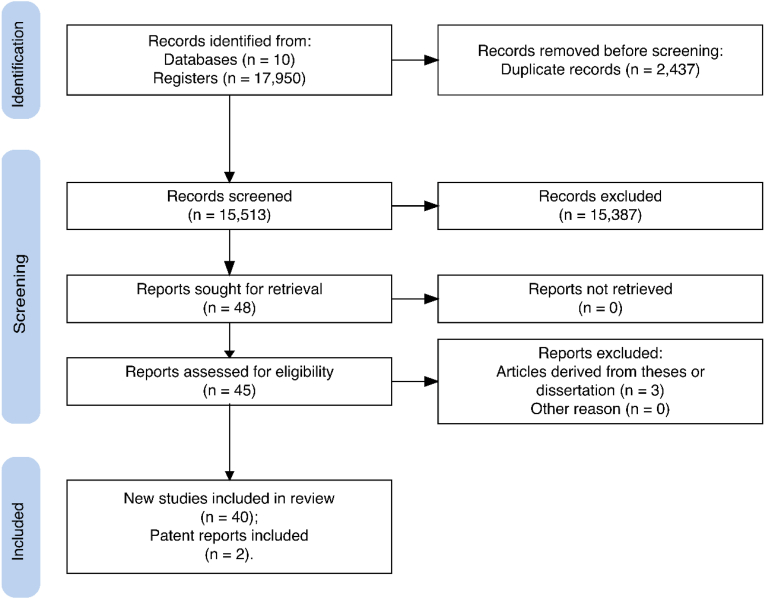
Flowchart showing the research design process of the study.

Google Scholar found 2,650 results; the BDTD 151 results; the Catálogo de Teses e Dissertações da CAPES, 396 results; PubMed, 24; the Virtual Health Library, 16; the Cochrane Library, 68; the NDLTD, 9,375; EBSCO, 62; and the REDIB, 138. Among these results, 40 different papers met the selection criteria for this review: 13 theses and dissertations, and 27 journal articles. INPI (https://busca.inpi.gov.br/pePI/jsp/patentes/PatenteSearchBasico.jsp) showed 31 results but only two of them were related to Amazon scorpions. An Espacenet search (https://worldwide.espacenet.com) showed that only 3 out of 38 results were indirectly related to Amazon scorpion venom. All papers retrieved from BDTD, CAPES, PubMed, the Virtual Health Library, and the Cochrane Library were found using Google. This proved to be the most effective search engine, covering more online scientific papers than any other search engine currently available (Martín-Martín et al., 2021). Fig. 1 shows a flow diagram detailing the selection process in the databases.

### 2.2 General information

#### 2.2.1 Scorpion species and publications

**Fig. 2:**
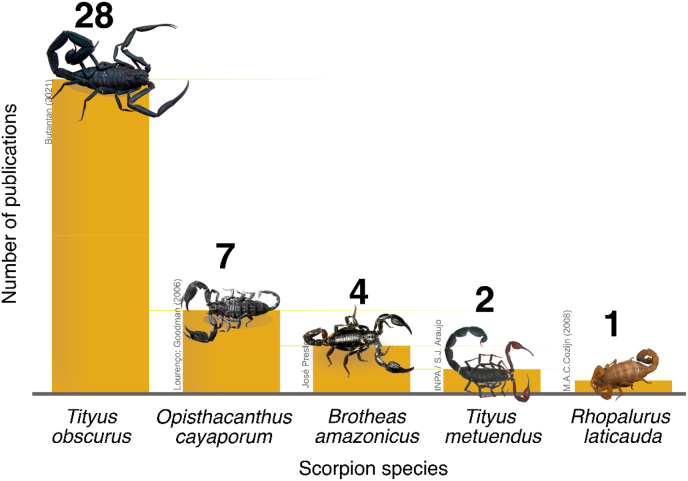
Number of studies on scorpion venom from the Brazilian Amazon from 2001 to 2021, by species. Since one study may cover several species, the sum does not reflect the total number of studies, n = 40.

The reviewed papers encompassed five different species, as presented in Table 2 and Fig. 2. The species *Tityus obscurus* was referred to by its synonym *T. cambridgei* in 11 studies. The species *Rhopalurus laticauda* was referred to by its synonym *R. crassicauda*. Over the last two decades, about 10% of scorpion species from the Brazilian Amazon region had their venoms studied, with two species being medically important. The most studied species is *T. obscurus,* which has also been observed by Martins and collaborators (2021).

**Table 2:** Scorpions from the Brazilian Amazon whose venoms were studied from 2001 to 2021 and their references.

Scorpion species	Reference
*B. amazonicus*	Higa (2008); Ireno (2009); Higa et al. (2014), Higa (2020)
*O. cayaporum*	Camargos (2009); Schwartz et al. (2008); Silva (2008); Silva et al. (2009); Camargos et al. (2011); Schwartz et al. (2013); Guilhelmelli et al. (2016).
*R. laticauda*	Abreu et al. (2020).
*T. metuendus*	Batista et al. (2018), Higa (2020).
*T. obscurus*	Mourão (2016); Batista et al. (2004); Batista et al. (2002a); Batista et al. (2002b); Borja-Oliveira et al. (2009); Carvalho (2017); Chang (2016); Da Mata et al. (2020); De Holanda and Júnior (2019); De Oliveira et al. (2018); de Paula Santos-da-Silva et al. (2017); Dias (2016); Dias et al. (2018); Duque et al. (2017); Ferreira and Carvalho (2017); Guerrero-Vargas et al. (2012); Guilhelmelli et al. (2016); Grottesi et al. (2003); Huang (2004); Liu and Lin (2003); Marques-Neto et al. (2018); Murgia et al. (2004); Pardal (2014); Simon et al. (2018); Stehling et al. (2012); Tibery et al. (2019); Veloso-Júnior (2019); Wang et al. (2009).

**Fig. 3:**
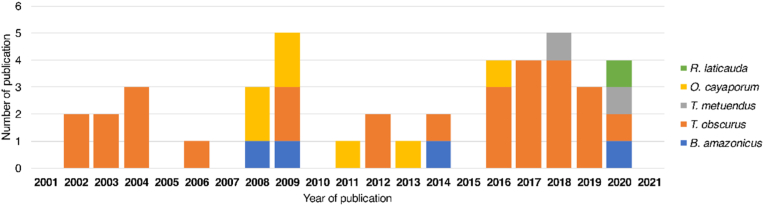
Studies on scorpion venom from the Brazilian Amazon grouped by year of publication.

The studies were published homogeneously throughout the period considered in this review, as shown in Fig. 3.

#### 2.2.2 Study objectives

**Fig. 4:**
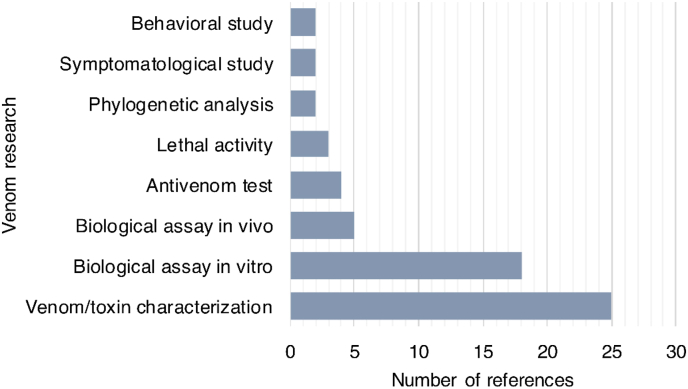
Number of studies on scorpion venom from the Brazilian Amazon from 2001 to 2021, classified by objectives. (Since one study may have multiple objectives, the sum does not reflect the total number of studies, n = 40).

**Fig. 5:**
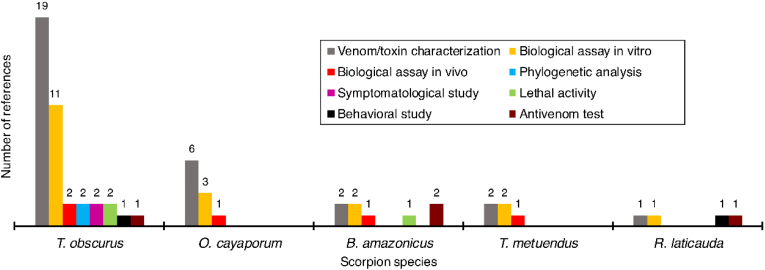
Number of different objectives of studies on scorpion venom from the Brazilian Amazon from 2001 to 2021 depending on the species in each paper. (Since one study can have multiple objectives, the sum does not reflect the total number of studies, n = 40).

The objectives of the studies could be divided into eight categories. Fig. 4, Fig. 5 show that most of the studies attempted to describe the molecular diversity of venoms from *T. obscurus, O. cayaporum*, *B. amazonicus*, *T. metuendus* and *R. laticauda* using biological assays. The only comprehensive study appears to be on *T. obscurus*, as it is one of the most medically important species in the region (Amado et al., 2021). All five species have been subjected to chemical characterization of their venom (e.g. Abreu et al., 2020; Batista et al., 2018; Camargos, 2009; Dias et al., 2018; Higa, 2008).

#### 2.2.3 Material origin

**Fig. 6:**
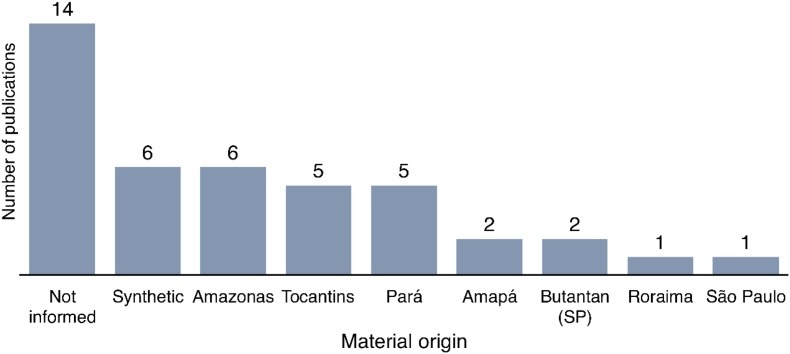
Absolute frequencies of studies on scorpion venom from the Brazilian Amazon from 2001 to 2021, grouped by origin of scorpions and toxins studied (n = 40).

Information regarding the collection sites of the scorpions could help to draw conclusions based on their distribution and guide future research. Table 1 shows that the toxin from the studied Amazonian scorpions came from Pará, collected in Santarém, Benevides, Belterra, Marajó Island, and Floresta Nacional do Tapajós. Some were also collected in Manaus (state of Amazonas), Palmas (Tocantins) and Boa Vista (Roraima). Five studies (12%) focused exclusively on synthetic toxins. Unfortunately, fourteen studies did not provide information on the origin of the scorpions and the toxins studied, representing 35% of the studies. Two studies (5%) used scorpions kept at Instituto Butantan in the state of São Paulo. Scorpions from Amazonas were collected in Manaus and other unknown locations. Fig. 6 shows the origin of the scorpions and toxins cited for each state. Table 3 provides details on the origin of the specimens and their associated toxins for each species.

**Table 3:** Scorpions from the Brazilian Amazon studied from 2001 to 2021, including the origin of the specimens and their toxins. (Since one study may cover several species, the sum does not reflect the total number of studies, n = 40).

Scorpion	Origin (Number of studies)
*B. amazonicus*	Amazonas (1); Not informed (2)
*O. cayaporum*	Tocantins (5); Not informed (1); Synthetic (1)
*R. laticauda*	Roraima (1)
*T. metuendus*	Amazonas (1)
*T. obscurus*	Amazonas (2); Amapá (2); Pará (5); Not informed (11); Synthetic (5); Instituto Butantan (2)

#### 2.2.4 Study location, funding and partnerships

Brazilian research institutions were in charge of 70% of the studies on Amazonian scorpion venom. These institutions received funding from various sources, including from other institutions in Brazil (28 studies), Mexico (2 studies), and Belgium (2 studies). Partnerships were also formed with research institutions from several countries, including 11 collaborations in Brazil, 3 in Mexico, and 1 each in Belgium, Italy, Germany, Colombia, the United States, and the United Kingdom. Three studies did not report funding sources, and 10 studies did not report any partnerships.

While Brazilian research institutions dominated, foreign institutions accounted for 27% of the studies on Amazonian scorpion venom. These studies were primarily conducted by institutions from Mexico (4 studies) and Taiwan (4 studies), with contributions from institutions in Italy (1 study) and the United Kingdom (1 study). Foreign funding for these studies came from different countries, including Mexico (3 studies), the United States (2 studies), Taiwan (1 study), and the United Kingdom (1 study). However, five studies did not report funding sources, and four studies did not report any partnerships.

### 2.3 Knowledge of scorpion species

In this section, we present the most extensively Amazonian scorpion species described in the literature during the study period. In the following sections, we will describe and highlight the main characteristics observed in each Amazonian species studied.

#### 2.3.1 *Tityus obscurus* Gervais, 1843

*T. obscurus* (family Buthidae), also known as *T. cambridgei*, *T. paraensis* and *T. amazonicus*, is a species of significant medical importance (Pardal, 2014). Adults are black, while juveniles have light spots. Its venom has been extensively studied, including toxin characterization, electrophysiological characterization, phylogenetic and structural analysis of the toxins, lethal activity analysis, antimicrobial, cytotoxicity and retroviral evaluation, molecular cloning and sequencing.

According to the reviewed literature, the venom and toxins of *T. obscurus* are complex and can help improve and understand scorpionism treatment, antivenoms and epidemiology for the Amazonian population. *T. obscurus* venom triggers a complex mechanism of envenoming pathogenesis. Moreover, studies on *T. obscurus* venom help to improve the treatment of diseases affecting the nervous and muscular systems, as well as infections caused by retroviruses, fungi and mycobacteria, and other diseases caused by enzymes, idiopathic pulmonary fibrosis, and enhance immunity. Due to its toxin activity, the venom has potential for treatments targeting neurotransmitter release, hormone secretion, regulation of fluid secretion and lymphocyte activation. The promising research on *T. obscurus*’ venom can potentially aid in the development of treatments for a wide range of diseases, from neurological and muscular system disorders to infections.

Batista and colleagues were the first to study and chemically characterize its venom. They discovered the following toxins: Tc48a, Tc49a, Tc49b, and Tc54 which recognize sodium channels (Batista et al., 2002a). They also isolated and described the toxins Tc30 and Tc32 as potent suppressors of potassium currents in human T lymphocytes (Batista et al., 2002b). In 2004, this group identified and described 26 sodium channel toxins (Tc1, Tc27, Tc29-33, Tc35, Tc37, Tc39-41, Tc43, Tc46, Tc48a, Tc48b, Tc49a, Tc49b, Tc50, Tc54, Tc56, Tc58, Tc61, Tc64, Tc66, and Tc83). Murgia et al. (2004) reported that Tc48b affects sodium permeability in pituitary GH3 cells. The toxin Tc54 was renamed To4 by Duque et al. (2017) after being electrophysiologically characterized as exhibiting a beta-type effect on different human sodium channel isoforms, exhibiting a beta-type effect on these channels. Liu & Lin (Liu and Lin, 2003) observed that Tc1 prefers the Kv1.1 potassium channel due to stronger electrostatic and hydrophobic interactions. Wang et al. (2009) demonstrated that a synthetic version of Tc1 has the same functional properties as the natural toxin, being a stable potassium channel blocker. Grottesi et al. (2003) studied the flexibility of Tc1 and found that it shares a common fold with agitoxin-2 and charybdotoxin from the scorpion *Leiurus quinquestriatus* var. *hebraeus*.

**Fig. 7:**
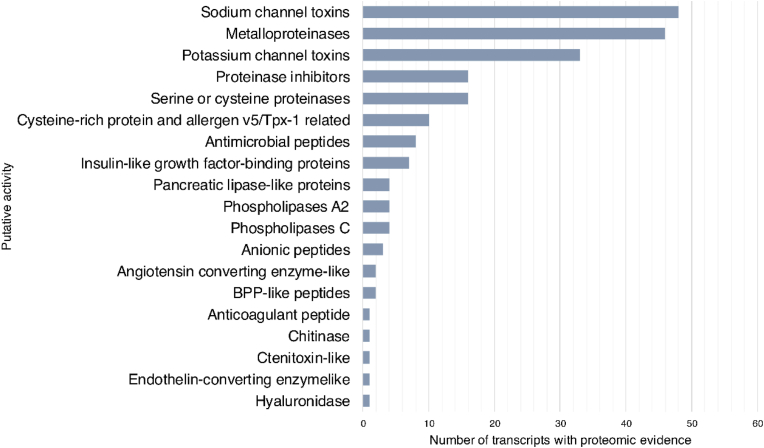
List of putative bioactive proteinaceous components observed by proteomics and transcriptomics in the two scorpions *Tityus obscurus* and *T. serrulatus*, adapted from the data provided in De Oliveira et al. (2008).

Guerrero-Vargas et al. (2012) isolated 15 sodium channel toxins (To1–To15), noting multiple names for the same toxins in the literature. They hypothesized a substantial cladistic difference between toxins produced by congeneric scorpions in south-eastern South America and those produced by northern Amazon basin scorpions. The authors also demonstrated that the alpha-class NaScTxs are closely related to Tpa4, Tpa5, Tpa6, To6, To7, To9, To10, and To14, whereas the beta-class NaScTxs are more closely related to Tpa7, Tpa8, To4, To8, To12, and To15 sequences. To5 may be an arthropod-specific toxin. In *T. obscurus* venom, Dias (2016) detected 517 peptides (27 sequenced) and 46 other non-peptidic compounds. Four peptides exhibited hemolytic activity. Tibery et al. (2019) isolated the beta-toxins Tc48b (or Tc49a) and Tc49b. Tc49b could inhibit most sodium channel isoforms, thereby altering the open probability during activation and steady-state inactivation in human cells. Dias et al. (2018) detected 27 peptides ranging from 400 to 4,000 Da in *T. obscurus*. Thirteen were biologically tested and caused hemolysis, as well as lactate dehydrogenase release from the mast cell cytoplasm into the surrounding environment. This potentiated significant inflammatory processes and changes in locomotion and lifting capacity. Huang (2004) and Chang (2016) studied the alpha-toxin Tc32 and demonstrated its inhibition of Shaker B and Kv1. x channels and described how the interaction occurs. Stehling et al. (2012) compared Tc32 with the TdK2 and TdK3 toxins. He hypothesized that the affinity and selectivity of the toxins are determined by differences in their electrostatic properties, contact surfaces and total dipole moment orientations. Fig. 7 shows the protein and peptide components observed by De Oliveira and collaborators (2018).

While the toxins shared similarities with some putative toxins found in other *Tityus* species, such as P84688, P84685, H1ZZH7, P60213, H1ZZIO, P60214, PO1496, H1ZZI3, H1ZZ12, and P60212, the authors emphasize that the *T. obscurus* venom components are not recognized by anti-*T. serrulatus* venom serum.

Interestingly, Pardal (2014) compared the venom of two *T. obscurus* populations from two regions of Pará and observed differences. The venom of western scorpions had more components than that of eastern scorpions, with more potassium and sodium modulators. This is why incidents in the western region of Pará are more severe than those in the eastern region. Additionally, the author recommends a taxonomic revision. This highlights that venom studies can significantly contribute to both species classification and phylogenetics.

Borja-Oliveira et al. (2009) observed in rat skeletal muscle that a 10 g/mL venom solution generated a gradual and sustained increase in contractile force for 120 min. Only higher concentrations promoted transient potentiation. They hypothesized that venom could be used clinically. Additionally, de Paula Santos-da-Silva et al. (2017) noted that rats showed signs of envenomation approximately 30 min after injection, with a peak of systemic effects 60 min after. This injection was performed using 0.1 mL/100 g of sodium chloride solution. Some of the effects observed were: hemorrhagic patches in the lung parenchyma and pleural regions at 10 mg/kg; also, extravasation of red blood cells in the parenchyma; decrease in general and locomotor activity at 60 min; breathing difficulty; piloerection; palpebral ptosis; excessive oral and nasal secretions; somnolence; photophobia; priapism; “wet dog shakes”, and immediate diuresis.

In biological tests from a biotechnological perspective, Marques-Neto et al. (2018) demonstrated that ToAP2 suppresses the growth of four *Mycobacterium massiliense* strains at 200 μM. It reduced the bacterial load in the liver, lung and spleen of mice, and recruits monocytes, neutrophils and eosinophils. Guilhelmelli et al. (2016) isolated and recorded that ToAP3, ToAP2-ToAP4, ToAcP, and NDBP-4.23 have antifungal properties against filamentous fungi and yeast such as *Candida*. Later, Ferreira & Carvalho (2017) isolated P42 (probably To4 or another) and demonstrated its antifungal activity against yeast strains: *C. albicans*, *C. tropicalis* and *C. parapsilosis*, and its antibacterial activity against *Escherichia coli* and *Staphylococcus aureus*. Da Mata et al. (2020) tested eight synthetic peptides and demonstrated that the P6 peptide has low cytotoxic activity against primary human leukocytes. It also has high antiretroviral activity against simian immunodeficiency virus replication in the HUT-78 cell line. De Holanda and Júnior (2019) observed that ToAP3 and ToAP4 can suppress inflammatory responses and modulate the activation and maturation of dendritic cells in mice, making them suitable candidates for anti-inflammatory therapies. Simon et al. (2018) also tested these toxins against early-stage idiopathic pulmonary fibrosis in rats and found that the toxins stabilized lung damage and slowed disease progression. Mourão (2016) isolated ToPI1 and synthesized it (ToPI1s). Its activity against trypsin in chromogenic assays and lack of adverse effects in mice make it a good candidate for therapeutic purposes.

#### 2.3.2 *Opisthacanthus cayaporum* Vellard, 1932

*O. cayaporum* (family Hormuridae) is a black scorpion from the south of Pará to the central region of Tocantins, reaching between 7 and 9 cm in length. It has no medical importance (Schwartz et al., 2008). Its venom underwent purification and characterization of its peptides, functional characterization and evaluation of its antifungal activities, and transcriptomic studies. Specimens were predominantly collected in Tocantins. Schwartz et al. (2008) detected 250 different components in the venom, including a peptide with 65% similarity to the α-KTx 6.10 toxin (OcKTx5). They suggested that the venom was insect-specific, harmless to mammals, and had phospholipase and antibacterial activity. Later, Schwartz et al. (2013) studied the OcyKTx2 peptide and described it as having 34 amino acids, four disulfide bridges, and a molecular weight of 3,807 Da. They compared it to other toxins and demonstrated that it acts on Shaker B and Kv1.3 channels at nanomolar concentrations.

Silva (2008) characterized scorpion venom gland transcripts by building a cDNA library with 67 distinct sequences. This library included toxin-like sequences and others involved in gene and protein expression. The peptide Cayaporina (NDBP 3.7) exhibited antimicrobial activity against *E. coli* and *S. aureus*, with no hemolytic activity in human erythrocytes. Camargos (2009) identified the potassium channel blocker κ-KTx 2.5 (3 kDa), and partially sequenced a Scorpine-like and a non-disulfide bridged peptide (NDBP) OcCT2f, which showed antimicrobial activity and warrants further investigation. The peptide κ-KTx 2.5 was later investigated by Camargos et al. (2011) and had no effect on *E. coli* and *S. aureus* at 128 mM. Guilhelmelli et al. (2016) studied the effects of three peptides from *O. cayaporum* as antifungals: Con10 (27 amino acids long), NDBP-5.7 (13 amino acids), and NDBP-5.8 (14 amino acids). Con10 showed antifungal activity, particularly against *Candida albicans*. NDBP-5.7 and NDBP5.8 displayed activity against *C. albicans* and *C. tropicalis*.

#### 2.3.3 *Brotheas amazonicus* Lourenço, 1988

*B. amazonicus* (family Chactidae) is a black scorpion with reddish tips and telson (Martins et al., 2021), found in Amazonas, Roraima, and Rondônia, and known for its low lethality venom. Its venom was subjected to molecular characterization, biological activity analysis and evaluation for potential biotechnological uses. Higa (2008) demonstrated that its venom does not induce bleeding or blood coagulation in mice. This confirmed its low toxicity and that its toxins have potent analgesic activity against inflammatory pain, suggesting potential value for analgesic drug development. The author also found that its venom exhibits phospholipase A_2_ activity. He suggests that its 7080 Da serine proteases are responsible for the proteolytic activity. Higa et al. (2014) also demonstrated that the venom can degrade bovine fibrinogen without fibrin clot formation. This makes it a potential candidate for antithrombotic drugs and vaccines against scorpion envenomation. Ireno (2009) identified 201 molecular species, including peptides ranging from 0.8 to 17 kDa, and sequenced eight peptides.

#### 2.3.4 *Tityus metuendus* Pocock, 1897

*T. metuendus* (family Buthidae) is a medically significant species from the Amazon. It has a reddish-black coloration. Batista et al. (2018) demonstrated that the venom collected in Manaus (Amazonas) is highly toxic to mammals and lethal to mice even at low concentrations. The venom contains alpha and beta-toxins closely resembling those found in *T. obscurus*. Among the various proteins and peptides, the authors aim to identify sodium and potassium channel toxins, hyaluronidases, metalloproteinases, endothelin, and angiotensin-converting enzymes, allergens, and bradykinin-potentiating peptides in the venom. This study highlights the need for further research.

#### 2.3.5 *Rhopalurus laticauda* Thorell 1876

*R. laticauda* (family Buthidae) is found in Roraima, south of Guyana and Venezuela, in deciduous forests and semi-arid regions. This species ranges in size from 45 to 70 mm, has a yellowish-brown coloration with a dark tail, and can be found under rocks, tree barks and fallen logs (Martins et al., 2021). Abreu et al. (2020) conducted a comprehensive study of its venom, using samples from Boa Vista, the capital of Roraima.

They isolated the major toxin Rc1**,** weighing about 6.5 kDa. This toxin represented 24 percent of the total protein of the soluble crude venom and was classified as a beta-neurotoxin. The crude venom could not be recognized by Brazilian antivenoms. However, a fraction of the venom containing hyaluronidase was recognized by the general arachnid antivenom. It was found to be specific to mammalian and insect voltage-gated sodium channels and exhibited cytotoxic effects and strong pro-inflammatory activities.

### 2.4 Patent application

Despite the vast biodiversity of scorpions in the Amazon, research has primarily focused on the venom of the *T. obscurus* species in the context of patent applications (Table 4). These applications pertain to the antimicrobial peptide and trypsin inhibitor activities of the species and are typically owned by universities and research institutes (De Marco Almeida et al., 2015). Other inventors have looked into the possibility of knotting peptides derived (Table 5) from known entities, including toxins or proteins associated with venom. Among these, the Amazonian scorpion *T. obscurus* has been identified as a source, with applications ranging from therapeutic agents against cartilage disorders.

**Table 4:** Patents involving the venom or toxin of *Tityus obscurus*.

Toxin	Type of invention	Priority number Priority date Reference	Applicants	IPC
ToAP2	Antimicrobial peptide	BR102017024728A2 2017-11-17	FUB; União Brasileira de Educação Católica [BR]	A61 C07 C12
ToPi1s, ToPi1-k21a, cToPi1s, cToPi1-k21a	Serine peptidase inhibitor	BR102017013362A2 2017-06-20	FUB; Universidad Nacional Autónoma de México; UFG; EMBRAPA [BR/MX]	A61 C07 G01

**Abbreviations:** IPC – International Patent Classification: A61: medical or veterinary science; hygiene; C07: organic chemistry; C12: biochemistry; beer; spirits; wine; vinegar; microbiology; enzymology; mutation or genetic engineering; EMBRAPA: Empresa Brasileira de Pesquisa Agropecuária; UFG: Universidade Federal de Goías. FUB: Fundação Universidade de Brasília. Source: Adapted from De Castro and collaborators (2022).

**Table 5:** Cartilage homing peptides that cited the venom of *T. obscurus* or toxins from this species.

Type of invention	Priority to/Priority date Publication date/publication of	Assigned to	ICP
Cartilage-homing peptides	EP16845226.6A/2016-09-09 WO2017044894A2/2017-03-16 WO2017044894A3/2017-05-11	Blaze Bioscience Inc Fred Hutchinson Cancer Center	A61K38/17
Conjugates of cartilage-homing peptides	US20210252159A1/2019-04-19 US20210252159A1/2021-08-19	Fred Hutchinson Cancer Center	A61K47/64
Cartilage-homing peptides	EP3347035A2/2018-07-18	Blaze Bioscience Inc Fred Hutchinson Cancer Center	A61K49/0056

**Abbreviations:** IPC – International Patent Classification: A61: medical or veterinary science; hygiene; A61K38/17: Peptides having more than 20 amino acids; Gastrins; Somatostatins; Melanotropins; Derivaives thereof from animals; from humans; A61K47/64 - Drug-peptide, drug-protein or drug-polyamino acid conjugates, i.e. the modifying agent being a peptide, protein or polyamino acid which is covalently bonded or complexed to a therapeutically active agent; A61K49/0056 - Peptides, proteins, polyamino acids.

The ToAP2 is a non-disulfide-bridged antimicrobial peptide (NDBP) derived through bioinformatics analysis of a cDNA library sourced from the venom gland of the scorpion *T. obscurus*. It has exhibited potent antimicrobial activity against *Mycobacterium massiliense* strains (GO01, GO06, GO08, and CRM0020) as reported by Trentini et al. (2017) and Marques-Neto et al. (2018). Moreover, this peptide has demonstrated antifungal properties against *Cryptococcus* spp. and *Candida albicans*, with Freitas et al. (2020) highlighting its efficacy at low concentrations and minimal toxicity to mammalian cells.

Another noteworthy patent invention stems from toxins found in *T. obscurus*, resulting in four peptides: ToPI1s, ToPI1-K21A, cToPI1s, and cToPI1-K21A. These peptides exhibit potent trypsin-inhibiting activity, originating from a modification of a scorpion venom peptide (Schwartz et al., 2020). ToPI1s and ToPI1-K21A consist of 33 amino acid residues, three e bonds, and C-terminal amidation. On the other hand, cToPI1s and cToPI1-K21A, through interaction with trypsin, adopt a cyclic structure with 32 residues and a Cys-Stabilized Alpha/Beta configuration, as shown by Mourão (2016) and Schwartz et al. (2020).

These peptides offer several advantages, including high chemical and thermal stability, lack of cytotoxicity in fibroblasts, low activity in potassium channels, and an absence of behavioral. These attributes render them attractive for diverse therapeutic applications, such as antiretrovirals, antitumor agents, or probes. According to Schwartz and collaborators (2020), the peptide ToPI1-K21A has been noted for its lower incidence of side effects when administered in mammals.

Olso et al. (2017; 2018) and Hopping (2019), along with their collaborators, proposed a pharmaceutical composition and method to target drug delivery to a specific region through a knotted peptide (Table 5), which may be a variant peptide belonging to a family member derived from different organisms, including *T. obscurus*.

## 3 Discussion

Martins et al. (2021) highlight the scarcity of research on scorpion venom in the Brazilian Amazon before 2001, with only one study in 2000. This suggests that interest in Amazonian scorpion venom is a recent phenomenon. Although extensive biochemical studies have been conducted since the early 21st century, it is still remarkable that only a limited number of species have been studied in the vast Amazonian biome. The research on scorpion venom in the Brazilian Amazon is still in its early stages, thus providing ample opportunities for further exploration. Despite the wealth of known pharmacological properties associated with scorpion venom and its toxins, these properties are largely unexplored in the specific context of the Brazilian Amazon. Promising avenues for investigation include untapped areas such as antiosteoporotic, antimalarial, anti-inflammatory, antiepileptic, analgesic, antineoplastic, and other potential therapeutic properties (Ahmadi et al., 2020; Ghosh et al., 2019).

Based on the comprehensive description and discussion provided thus far, along with the data presented in Table 6, Table 7, it is evident that scientists have achieved remarkable advancements. Though progress has been made in understanding scorpion venom biochemistry in Brazil and the Amazon, much remains unexplored due to the region's vast biodiversity. The use of scorpion venom in biotechnology offers the potential for medical and environmental breakthroughs. Studying a wider range of Amazonian scorpion species could lead to groundbreaking discoveries. Biotechnological exploration of scorpion venom has uncovered treatments for various conditions. Specifically, scorpion venom shows promise in the treatment of muscular and neurological disorders, thrombosis, and the development of region-specific anti-scorpion serums tailored to Amazonian venom profiles. Scorpion venom has great potential as a valuable resource to address medical challenges specific to the Amazon region.

**Table 6:** Main venom properties and potential biotechnological applications of Brazilian Amazonian scorpions.

Scorpion	Physiological properties of the venom	Potential biotechnological application
*T. obscurus*	Hemorrhagic patches in the lung parenchyma (de Paula Santos-da-Silva et al., 2017); Edematogenic and moderate nociceptive activity (de Paula Santos-da-Silva et al., 2017); No pulmonary edema in mice (de Paula Santos-da-Silva et al., 2017); Immobility, piloerection, breathing and locomotion difficulty, somnolence, photophobia, priapism, “wet dog shakes”, immediate diuresis (de Paula Santos-da-Silva et al., 2017); Lethal dose: 3.13 mg/kg (de Paula Santos-da-Silva et al., 2017); Uncomfortable pain and inflammatory effects (Dias et al., 2018); Increased twitching (Borja-Oliveira et al., 2009); Not recognized by *T. serrulatus* antivenom (De Oliveira et al., 2018)	Muscle strength improvement (Borja-Oliveira et al., 2009); Neurological treatments (Borja-Oliveira et al., 2009). *T. obscurus* antivenom (De Oliveira et al., 2018);
*T. metuendus*	Restlessness, piloerection, sialorrhea, hyperactivity, respiratory difficulties, paralysis of limbs, exophthalmos, loss of equilibrium, convulsions and death in mice (Batista et al., 2018). Toxic to mammals (Batista et al., 2018).; Affects hNav 1.3 the most (Batista et al., 2018); Bradykinin-potentiating (Batista et al., 2018).	–
*R. laticauda*	Induces hypernociceptive response in mice (Abreu et al., 2020); Increases IL6 production (Abreu et al., 2020); Not recognized by Brazilian antivenoms (Abreu et al., 2020).	*R. laticauda* antivenom (Abreu et al., 2020);
*O. cayaporum*	APs reduction of cockroach nerve chord at 22 mg/ml (Schwartz et al., 2008).	–
*B. amazonicus*	Low toxicity to humans (Higa, 2008); Phospholipase A2 activity (Higa, 2008); Serine proteases ranging from 70 to 80 kDa giveoteolytic activity to the venom (Higa, 2008); Neutralized by *T. serrulatus* antivenom (Higa, 2008); Degrades Aα and Bβ subunits of fibrinogen (Ireno, 2009).	Human pharmaceuticals (Higa, 2008); Analgesic drugs (Higa, 2008); Antithrombotic drugs (Ireno, 2009).

**Table 7:** Main properties of the toxins and potential biotechnological applications of Brazilian Amazonian scorpions. Scorpions: *Oc = O. cayaporum; To = T. obscurus; Rl = R. laticauda.*

Toxin	Scorpion	Properties	Biotechnological application tested
**Con10**	*Oc*	-	Antifungal against *Candida. albicans* (Guilhelmelli et al., 2016)
**NDB-4.23**	*To*	1733.0 Da (da Mata et al., 2020);	
**NDBP 3.7**	*Oc*	4675 Da (Silva, 2008); no hemolytic activity008); positive antibiotic activity (Silva, 2008).	Antibiotic against *Staphylococcus aureus* and *Escherichia coli* (Silva, 2008)
**NDBP-5.5**	*Oc*	1332.0 Da (da Mata et al., 2020);	
**NDBP-5.7**	*Oc*	1434.0 Da (da Mata et al., 2020);	Antifungal against *Candida. albicans*, *C. tropicalis* (Guilhelmelli et al., 2016)
**NDBP-5.8**	*Oc*	1512.86 Da (da Mata et al., 2020).	Antifungal against *Candida. albicans*, *C. tropicalis* (Guilhelmelli et al., 2016)
**OcCT2f**	*Oc*	1511.913 Da (Camargos, 2009); antimicrobial activity (Camargos, 2009);	Antibiotic (Camargos, 2009);
**Ocy37.75**	*Oc*	Phospholipase activity (Schwartz et al., 2008).	
**Ocy39.10**	*Oc*	Phospholipase activity (Schwartz et al., 2008).	
**Ocy39.87**	*Oc*		Antibiotic against *Staphylococcus aureus* (Schwartz et al., 2008)
**OcyC10**	*Oc*		
**OcyC7**	*Oc*		Potential antimicrobial and antiparasitic (Silva et al., 2009)
**OcyC8**	*Oc*	3136.36 Da (Camargos, 2009); Csα/α fold family (Silva et al., 2009). Potassium channel toxin kappa-KTx 2.5 (Camargos et al., 2011)	
**OcyKTx2**	*Oc*	3875 Da (Schwartz et al., 2013). Reversibly blocks Shaker B potassium-channels (expressed in insect Sf9 cells) with a Kd of 96.6 nM, and presents an even better affinity toward hKv1.3 (KCNA3), blocking it with a Kd of 17.7 nM (Schwartz et al., 2013); inhibits the proliferation of effector memory T cells in humans and rats (Schwartz et al., 2013)	
**Rc1**	*Rl*	Sodium-channel toxin (Abreu et al., 2020); induces hypernociceptive response in mice (Abreu et al., 2020); stimulates TNF-α production (Abreu et al., 2020); potent inflammatory toxin (Abreu et al., 2020).	
**Tc1**	*To*	Potassium-channel peptide (Grottesi et al., 2003; Liu and Lin, 2003)); Tc1 binds preferentially towards Kv1.1 than KcsA due to the stronger electrostatic and hydrophobic interactions (Liu and Lin, 2003)	
**Tc30**	*To*	3871.8 Da (Batista et al., 2004); α-KTx4.4 (Batista et al., 2002a); *Shaker* B potassium channels blockage (Batista et al., 2002a)	
**Tc32**	*To*	3521.5 Da (Batista et al., 2004); *Shaker* B potassium channels blockage (Batista et al., 2002a; Chang SF, 2016; Huang, 2004).	
**Tc41**	*To*	7109.4 Da (Batista et al., 2004);	
**Tc43**	*To*	7266.0 Da (Batista et al., 2004);	
**Tc48a**	*To*	7319.3 Da, sodium-channel peptide (Guerrero-Vargas et al., 2012);	
**Tc48b/Tc49a**	*To*	7385.4 Da, sodium-channel peptide (Guerrero-Vargas et al., 2012);	
**Tc50**	*To*	7073.0 Da (Batista et al., 2004);	
**Tc54**	*To*	7253.2 Da (Batista et al., 2004);	
**Tc66**	*To*	6935.3 Da (Batista et al., 2004);	
**To1/Tc49b**	*To*	7400.2 Da; beta-toxin activity on VdNa_V_1 and BgNa_V_1; reduces the peak of Na + currents [48]; 7404.5 Da, *bonafide* sodium-channel peptide [27, 36]; it produces excitability, respiratory problems, convulsions, death in mice (Batista et al., 2002a, Batista et al., 2002b).	
**To10**	*To*	6940.9 Da, sodium-channel peptide (Guerrero-Vargas et al., 2012);	
**To11**	*To*	7154.2 Da, sodium-channel peptide (Guerrero-Vargas et al., 2012);	
**To12**	*To*	7171.2 Da, sodium-channel peptide (Guerrero-Vargas et al., 2012);	
**To13**	*To*	8054.0 Da, sodium-channel peptide (De Oliveira et al., 2018; Guerrero-Vargas et al., 2012);	
**To14**	*To*	7953.0 Da, sodium-channel peptide (Guerrero-Vargas et al., 2012);	
**To15**	*To*	7195.1 Da, sodium-channel peptide (Guerrero-Vargas et al., 2012);	
**To27/Tc27**	*To*	1075 Da (Dias et al., 2018)/4103.1 Da (Batista et al., 2004);	
**To29/Tc29**	*To*	917 Da (Dias et al., 2018)/4150.3 Da (Batista et al., 2004);	
**To31/Tc31**	*To*	866 Da (Dias et al., 2018)/4304.4 Da (Batista et al., 2004);	
**To33/Tc33**	*To*	1179 Da (Dias et al., 2018)/3807.9 Da (Batista et al., 2004);	
**To35/Tc35**	*To*	995 Da (Dias et al., 2018)/3926.2 Da (Batista et al., 2004);	
**To37/Tc37**	*To*	1159 Da (Dias et al., 2018)/7265.6 Da (Batista et al., 2004);	
**To39/Tc39**	*To*	1141 Da (Dias et al., 2018)/2744.1 Da (Batista et al., 2004);	
**To4**	*To*	7249.44 Da. Inhibits mammalian and insect's sodium channels. Related to beta-NaScTxs (Duque et al., 2017).	Antifungal against *Candida. albicans*, *C. tropicalis* and *C. parapsilosis* (Carvalho, 2017)
**To40/Tc40**	*To*	1049 Da (Dias et al., 2018)/7796.4 Da (Batista et al., 2004);	
**To46/Tc46**	*To*	1113 Da (Dias et al., 2018)/6032.0 Da (Batista et al., 2004);	
**To5**	*To*	6937.7 Da, sodium-channel peptide (De Oliveira et al., 2018; Guerrero-Vargas et al., 2012);	
**To56/Tc56**	*To*	1114 Da (Dias et al., 2018)/7299.0 Da (Batista et al., 2004);	
**To58/Tc58**	*To*	1132 Da (Dias et al., 2018)/5504.1 Da (Batista et al., 2004);	
**To6**	*To*	7266.3 Da, sodium-channel peptide (Guerrero-Vargas et al., 2012);	
**To61/Tc61**	*To*	1079 Da (Dias et al., 2018)/7105.0 Da (Batista et al., 2004);	
**To64**	*To*	1127 Da (Dias et al., 2018)/7628.7 Da (Batista et al., 2004);	
**To7**	*To*	7074.1 Da, sodium-channel peptide (Guerrero-Vargas et al., 2012);	
**To8**	*To*	7050.0 Da, sodium-channel peptide (Guerrero-Vargas et al., 2012);	
**To83/Tc83**	*To*	1159 Da (Dias et al., 2018)/25402.0 Da (Batista et al., 2004);	
**To9**	*To*	7155.2 Da, sodium-channel peptide (Guerrero-Vargas et al., 2012);	
**ToAcP**	*To*	2757.0 Da (da Mata et al., 2020);	
**ToAP2**	*To*	3000.7 Da (da Mata et al., 2020); antiretroviral (da Mata et al., 2020) and antibiotic (Marques-Neto et al., 2018) activity.	Antiretroviral (da Mata et al., 2020); Anti-*Mycobacterium massiliense* (Marques-Neto et al., 2018);
**ToAP3**	*To*	1700.1 Da (da Mata et al., 2020); toxic at 50 μM (Veloso-Júnior, 2019); reduce TNF-α secretion (Veloso-Júnior, 2019);	Idiopathic pulmonary fibrosis treatment (Simon et al., 2018). Anti-*Cryptococcus neoformans* (Veloso-Júnior, 2019)
**ToAP4**	*To*	1762.1 Da (da Mata et al., 2020); toxic at 50 μM (Veloso-Júnior, 2019); reduce TNF-α secretion (Veloso-Júnior, 2019);	Idiopathic pulmonary fibrosis treatment (Simon et al., 2018). Anti-*Cryptococcus neoformans* (Veloso-Júnior, 2019).
**ToPI1s**	*To*	3756.2 Da; potent trypsin inhibitory activity (Mourão, 2016)	CD4 cells stimulation against HIV (Mourão, 2016).
**κ-KTx2.5**	*Oc*	Sodium-channel blockage at high concentrations (Camargos, 2009). No antibacterial activity (Camargos, 2009).	

While highly venomous scorpions often receive considerable attention for their potential harm to humans, harmless scorpion species are also highly valuable for scientific research. One such example is *Brotheas amazonicus*. This species has been studied even though it poses no threat to humans. In their work, Ward et al. (2018) emphasize the continued importance of studying harmless scorpions for drug development and medicinal purposes. These harmless scorpion species may represent an untapped source of valuable compounds and medicines.

The distribution of funding and collaborations highlights the predominantly national focus of Brazilian research institutions in the study of Amazonian scorpion venom. Nevertheless, the involvement of foreign institutions from seven different countries demonstrates the international interest and collaboration in this field. Particularly noteworthy is the extensive study of *Tityus obscurus*, which has garnered considerable attention from the global scientific community. The recognition of *T. obscurus* venom as a valuable resource for scientific exploration and potential biotechnological applications is highlighted by the interest of researchers globally.

The specimens *T. silvestris* and *T. apiacas* are of medical interest, but there is a lack of studies on the epidemiological and clinical manifestations of the accidents (Gomes et al., 2020; Coelho et al., 2016; Monteiro et al., 2019). Furthermore, there is no information on venom chemical characterization or therapeutic application based on scorpion venom during the investigation period. Martins and collaborators (2021) argue that more studies are needed to fill the gaps in the knowledge of Amazon scorpions, with a focus on the chemical composition, biological aspects, epidemiological and clinical characterization of scorpion accidents in Brazil.

Documenting the collection sites of scorpions is crucial for venom studies. This data helps researchers understand the distribution of scorpions and determine future research areas. By identifying the exact regions where scorpions are located, researchers can gain valuable insights into their habitats, ecological preferences, and potential variations in venom composition among different populations. Understanding the geographical origins of the scorpions studied allows researchers to consider regional differences in venom composition and potentially correlate these variations with ecological factors, including habitat, altitude, or climatic conditions. This data helps develop targeted studies and improves our understanding of the diverse venom profiles of scorpions in different regions.

Some studies have focused exclusively on synthetic toxins, indicating a distinct area of research where synthetic compounds are designed and investigated for their venom-like properties. However, there is a worrying lack of information as twelve studies (32% of the total) did not specify the origins of the scorpions or the toxins examined. This lack of information may hinder the ability to establish precise links between venom characteristics and their geographical context. Providing complete and transparent information regarding specimen origin is crucial for reproducibility, comparability, and advancing our understanding of scorpion venoms.

Two studies (5.4%) utilized scorpions kept at Instituto Butantan in the state of São Paulo. This suggests the use of captive scorpions, which allows for controlled laboratory studies. Although captive scorpions may not represent the full range of venom profiles found in the wild, they allow for controlled experiments, comparative analysis, and targeted studies on captive-bred individuals.

Documentation of the collection sites is therefore critical in scorpion venom studies, as it allows researchers to gain valuable insights to understand regional variations for instance, and guide future research. However, there is a need for improved reporting standards, as a significant portion of the studies did not provide information on the origin of the scorpions and toxins studied. By addressing these issues and ensuring transparent reporting, researchers can improve the reproducibility and comparability of studies, ultimately advancing our understanding of the diverse and complex world of scorpion venoms.

Advancing research on scorpion venom in the Brazilian 10.13039/100022984Amazon requires overcoming funding and collaboration challenges (Ubfal and Maffioli, 2011). Securing enough funds is key to conducting comprehensive studies, purchasing equipment, building facilities, and obtaining research materials. Additionally, fostering collaboration among researchers and institutions is essential for pooling expertise, sharing resources, and addressing the complexities of scorpion venom research. These collaborative efforts facilitate the exchange of knowledge and provide access to a variety of scorpion species, resulting in synergistic research initiatives. Combining resources and expertise helps researchers overcome the challenges of the diverse Amazon biome, leading to impactful discoveries in scorpion venom research. Therefore, securing funding and fostering collaborations are essential for successful research in this critical area.

Addressing the scarcity of professionals in scorpion venom research requires a focus on education. The shortage of experts in this field can be partially attributed to the absence of specialized training programs specifically designed for scorpion venom studies. To improve our understanding of the intricate nature of scorpion venom and harness its potential for various applications, it is imperative to prioritize the development and expansion of educational initiatives.

Establishing dedicated academic programs and courses is crucial for providing aspiring researchers with the necessary knowledge and skills to navigate the complexities of scorpion venom research. Collaboration with academic institutions, research centers, and industry partners is needed to design curriculum modules that cover a wide range of disciplines, including biochemistry, pharmacology, and toxinology. By promoting a multidisciplinary approach through educational frameworks, future professionals can acquire the essential interdisciplinary expertise needed to address the multifaceted nature of scorpion venom research.

These efforts must be accompanied by public awareness of the importance of scorpion venom research. Educating the public on the potential medical, pharmaceutical, and biotechnological applications of scorpion venom can generate support for educational programs and research endeavors. Public engagement can also help to dispel misconceptions about the field and highlight its relevance to scientific and societal progress.

## 4 Conclusion

During the period considered in this review, only a few Amazonian scorpions from Brazil were studied for their venom. There is still a lot of research to be done in this area. While the venom of these scorpions holds significant potential for pharmaceutical and clinical applications, it remains mostly unexplored and poorly understood. The Amazon's rich biodiversity provides an opportunity to uncover novel bioactive compounds that can shed light on envenomation processes unique to the region. These compounds could not only improve the effectiveness of antivenoms but also contribute to the development of new technologies and therapeutics, in line with the goals of the Global One Health initiative.

Securing adequate funding and fostering collaboration between institutions are crucial to the success and continuity of research. The Amazon rainforest is home to a vast array of known and unknown species, many of which possess unique compounds that could be used for therapeutic purposes. Collaborating with other institutions and providing funding will help us identify and utilize these compounds in the treatment of venomous stings, while also developing novel technologies and therapies that can benefit global health.

## Funding

This work was supported by the Pro-Rectory of Research and Post-Graduation (PRPPG) of the Federal University of Roraima (UFRR), Edital 14/2022 and the Association Plateforme BioPark d’Archamps (France) for supporting part of this work through its research and development program.

## Ethics in publishing

We, Joel Ramanan da Cruz, Philippe Bulet and Cléria Mendonça de Moraes, are the authors of the manuscript entitled “Exploring the potential of Brazilian Amazonian scorpion venoms: a comprehensive review of research from 2001 to 2021”. In the common consensus, we have agreed for authorship, read and approved the manuscript submitted. Our agreement also includes the subsequent publication following approval by the Editors. We confirm that there is no conflict of interest.

This work does not involve the use of human or animal subjects, because it is based on secondary studies available in the literature. It is important to mention that we have cited the original source properly.

We have adhered to Good Publication Practices (GPP) and Good Science.

## CRediT authorship contribution statement

**Joel Ramanan da Cruz:** Writing - review & editing, Writing - original draft, Validation, Resources, Methodology, Formal analysis, Data curation, Conceptualization. **Philippe Bulet:** Writing - review & editing, Validation, Resources, Funding acquisition, Formal analysis. **Cléria Mendonça de Moraes:** Writing - review & editing, Validation, Supervision, Project administration, Methodology, Funding acquisition, Formal analysis, Data curation, Conceptualization.

## Declaration of competing interest

The authors declare the following financial interests/personal relationships which may be considered as potential competing interests:Cleria Mendonca de Moraes reports financial support was provided by Federal University of Roraima. Philippe Bulet reports financial support was provided by 10.13039/501100004794National Centre for Scientific Research. If there are other authors, they declare that they have no known competing financial interests or personal relationships that could have appeared to influence the work reported in this paper.

## Data Availability

No data was used for the research described in the article.
